# Fatal Injuries Following Aggressive Cardiopulmonary Resuscitation in a Young Woman

**DOI:** 10.7759/cureus.93860

**Published:** 2025-10-05

**Authors:** Lucia Ihnát Rudinská, Ilker Sengul, Demet Sengul, Jana Mertová, Peter Ihnát

**Affiliations:** 1 Forensic Medicine, University Hospital Ostrava, Ostrava, CZE; 2 Endocrine and General Surgery, Faculty of Medicine, Giresun University, Giresun, TUR; 3 Pathology, Faculty of Medicine, Giresun University, Giresun, TUR; 4 General Surgery, Faculty of Medicine, University of Ostrava, Ostrava, CZE

**Keywords:** aortic tear, cardiac arrest, coronary artery hypoplasia, cpr-associated injuries, forensic medicine, forensic pathology, histopathology, pathology, surgery, vital reaction to trauma

## Abstract

Cardiopulmonary resuscitation (CPR) is a complex of emergency procedures aimed at restoring vital functions in individuals experiencing cardiac arrest. Despite its life-saving potential, CPR can result in a range of injuries, from minor abrasions to severe, life-threatening trauma. This vignette case details the sudden death of a 44-year-old woman who suffered extensive intra-thoracic and intra-abdominal injuries during CPR. Although these injuries were severe, the immediate cause of death was acute coronary artery failure as a result of undiagnosed atherosclerosis and coronary artery hypoplasia. The woman collapsed at a social event and received telephone-assisted CPR from her intoxicated boyfriend, followed by professional resuscitation efforts. Autopsy revealed multiple rib fractures, sternum fracture, pleural hemorrhage, lung contusions, and aortic tear, alongside significant internal bleeding. The histopathological examination confirmed the presence of atherosclerosis and coronary artery hypoplasia, and, crucially, revealed the absence of a vital reaction at the margins of the traumatic injuries. This case highlights the paradoxical nature of CPR, where life-saving efforts can simultaneously cause significant harm. It also emphasizes the importance of considering CPR-related injuries during forensic examinations in order to determine the cause of death accurately. The findings contribute to the limited body of literature on fatal CPR-associated injuries and stress the need for awareness among medical professionals regarding the potential for such outcomes, particularly emphasizing the vital distinction between ante-mortem and post-mortem trauma.

## Introduction

Cardiopulmonary resuscitation (CPR) comprises a set of therapeutic interventions designed to save a person's life by restoring basic life functions, such as consciousness, breathing, and blood circulation. However, the process of trying to save a life often results in various injuries to the resuscitated person (known as CPR-associated injuries) [1,2]. These range from trivial injuries (skin abrasions and hematomas) to life-threatening injuries (cardiac tamponade, lung contusions/lacerations, laceration of intra-abdominal organs, etc.), thereby, in certain cases, injuries associated with CPR may not only harm the resuscitated person, but also completely nullify the effect of the CPR performed [2-5]. The present case details the sudden demise of a young woman who, prior to death, underwent CPR and sustained severe intra-thoracic and intra-abdominal injuries. While these injuries were classified as lethal, the immediate cause of death was ascertained to be acute coronary artery failure. This acute failure stemmed from the following two natural diseases affecting the cardiac vasculature: atherosclerosis and hypoplasia of the coronary arteries. Crucially, neither of these natural diseases had been diagnosed in the young woman during her lifetime. Given the limited data in the current literature regarding lethal injuries associated with CPR, the forensic pathologist performing an autopsy on a person with severe CPR-related injuries must consider these injuries as a potential cause of death or a significant contributing factor [6-11]. We present a case illustrating how severe, potentially lethal CPR-related injuries can occur even without the return of spontaneous circulation (ROSC), and discuss the critical forensic considerations necessary to differentiate post-mortem trauma from ante-mortem injury.

## Case presentation

Brief report

A 44-year-old woman was attending a village party where she consumed alcohol. Late in the evening, she abruptly collapsed, striking her face on the ground and immediately losing consciousness. The ambulance service was summoned, and telephone-assisted resuscitation (TANR) was initiated under the guidance of the dispatcher. TANR was performed by the woman’s boyfriend, who was later determined to have a blood alcohol level of 2.54 g/kg, indicating he was under the strong influence of alcohol. Upon the arrival of the ambulance, paramedics continued CPR. Initially, the woman was found to be in asystole. A peripheral venous line was secured, and the airway was secured with a connection for artificial pulmonary ventilation. Circulatory resuscitation involved the administration of epinephrine and Cordarone. Following defibrillation, the electrical activity of the heart was restored, presenting as bradycardia with the wide ventricular complexes. Afterward, the woman was transferred to the university hospital emergency department with ongoing resuscitation, where she subsequently died. Subsequently, the witnesses reported that the young woman collapsed while carrying beverages (beer and spirits) to the table. The medical records from her general practitioner indicated no history of monitored medical conditions. However, she had undergone sclerotherapy for small varicose veins in her lower limbs approximately one month before her death. The body was transported to the Department of Forensic Medicine at the University Hospital in Ostrava, Czech Republic, where a medical autopsy was conducted in accordance with Recommendation No. R (99) 3 on the Harmonization of Medico-Legal Autopsy Rules, adopted by the European Council in 1999 [12,13].

Summary of key events (timeline)

The timeline of events is as follows: (i) late evening - collapse occurred at a social event following alcohol consumption; (ii) immediate - a bystander, the intoxicated boyfriend, initiated telephone-assisted non-resuscitation (TANR) under dispatcher guidance; (iii) EMS arrival - professional paramedics continued CPR, with the initial rhythm noted as asystole; (iv) resuscitation efforts - administration of epinephrine and Cordarone was carried out, and defibrillation was performed; and (v) outcome - transient restoration of electrical activity was observed, presenting as bradycardia with wide ventricular complexes. The patient was transferred with ongoing resuscitation but subsequently died at the university hospital.

Autopsy and Histopathological Findings

**Figure 1:**
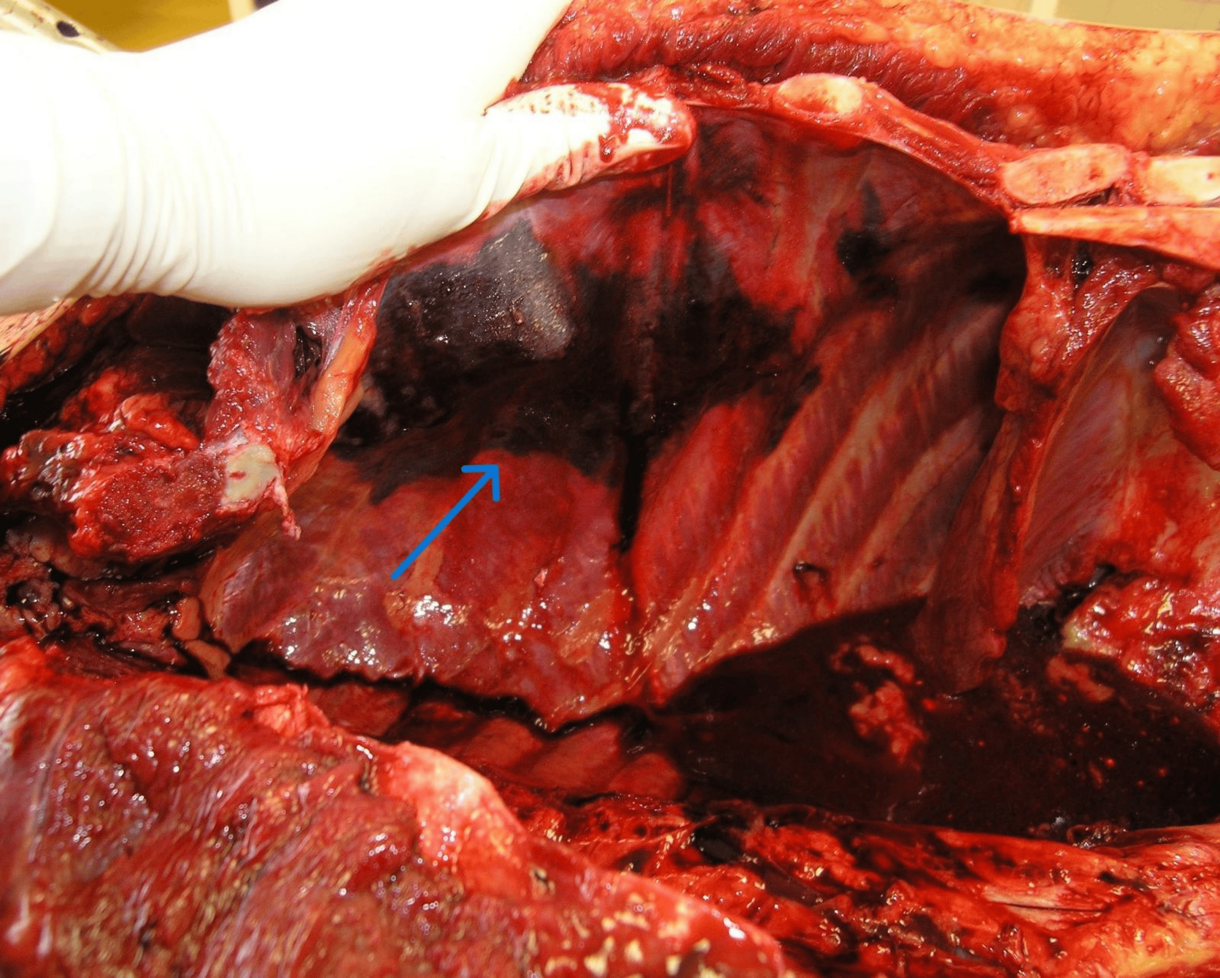
The pleural hemorrhage at the site of the serial rib fractures (blue arrow).

A standardized autopsy included both external and internal examination of the body, supplemented by routine histopathological sampling of major organs. The external post-mortem examination revealed superficial abrasions on the forehead, nose, and left knee, along with lacerations of the nose and upper lip. A superficial burn was observed on the central area of the chest, situated between the nipples. An injection mark was also present on the right elbow socket. The internal examination revealed bilateral serial rib fractures - fractures of the 2nd-7th ribs on the left side and fractures of the 2nd-4th and 6th-7th ribs on the right side, all found in the midclavicular line. A fracture of the sternum was also present. At the site of the serial rib fractures, there was massive pleural hemorrhage, accompanied by pulmonary contusions affecting both lung wings and hemorrhage in the mediastinal soft tissue (Figure 1).

**Figure 2:**
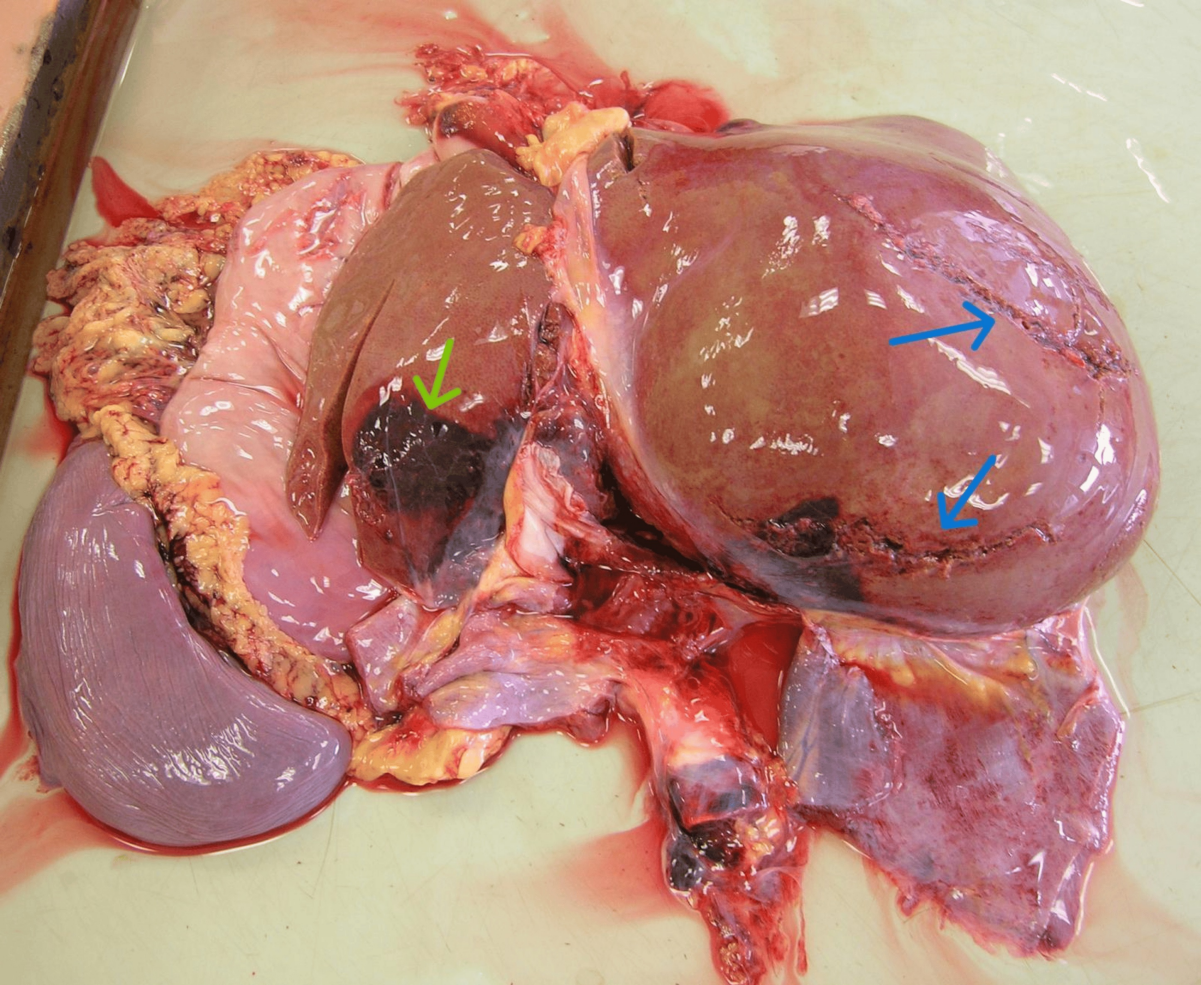
The rupture locations of the liver (the two blue arrows indicate the right lobe, while the yellow arrow indicates the left lobe).

Further findings included a contusion of the pericardium and a tear of the thoracic segment of the aorta located posterior to the origin of the left subclavian artery. Approximately 100 mL of liquid dark red blood was present in both the right and left pleural cavities. The abdominal cavity contained 400 mL of liquid blood, along with a rupture of the liver and small tears in the splenic hilum (Figure 2).

In addition to the traumatic injuries, the autopsy identified the following two natural diseases of the cardiovascular system: (i) atherosclerosis - small atherosclerotic plaques were found in the wall of the descending branch of the coronary artery, resulting in a narrowing of one quarter of the vessel, (ii) coronary artery hypoplasia (developmental defect) - a developmental defect was present, specifically hypoplasia of the right coronary artery and hypoplasia of the circumflex branch of the left coronary artery, with the lumen of these vessels measuring only 0.1 cm in diameter. A hemorrhage of the anterior papillary muscle of the left ventricle was also noted. Last but not least, the histopathology confirmed the atherosclerosis of the coronary arteries, signs characteristic of ischemic heart disease, pulmonary edema, the traumatic pulmonary contusions, fissures of the liver and spleen, and the rupture of the aorta.

Histopathological Examination of Trauma Margins

Microscopic examination of the edges of the aortic tear, hepatic ruptures, and pleural tissues demonstrated a definitive absence of features indicative of a vital reaction. Specifically, there was no evidence of acute inflammatory cell infiltration, endothelial proliferation, or organized thrombus formation at the sites of rupture or hemorrhage. This finding supports the determination that these severe traumatic injuries occurred subsequent to the cessation of circulation.

Based on the totality of the autopsy and histopathological findings, the cause of death was determined to be natural (non-traumatic). The basis for this conclusion rested on the presence of two natural diseases affecting the vasculature supplying the heart (atherosclerosis and coronary artery hypoplasia), neither of which had been diagnosed during the victim's lifetime, and the compelling histological evidence indicating the post-mortem nature of the CPR-associated trauma.

## Discussion

Definitely, cardiac arrest is an extreme emergency requiring the earliest possible initiation of CPR to save life, as irreversible changes in the central nervous system occur within minutes of this event. Cardiovascular disease is the leading cause of death in developed countries. The global incidence of out-of-hospital cardiac arrest is estimated at 62 cases per 100,000 people per year, with cardiovascular disease accounting for 75-85% of these arrests [14]. While the success rate of CPR typically ranges between 5% and 10%, bystander CPR significantly improves survival chances, approximately doubling them.

One of the crucial factors underlying the success of CPR is bystander CPR, which refers to CPR performed by a bystander witnessing a cardiac arrest. The chance of survival is approximately twice as high when CPR is initiated immediately by a bystander, an amateur rescuer [14,15]. Conversely, excessive (vehement) CPR can lead to severe and even lethal injuries in the patient. Indirect cardiac massage results not only in skeletal chest fractures but also in various injuries to the organs of the thoracic cavity. Observed injuries include hemorrhage, contusion, and laceration of the pleura and lungs; heart injuries (e.g., epicardial/myocardial hemorrhage, heart wall rupture, tamponade); diaphragm injuries; and injuries to the large vessels of the mediastinum [1-4,16]. Fatal injury to the ascending aortic segment due to CPR has been described in up to 1% of resuscitated cases. Bode and Joachim also describe instances of fatal injury to the ascending aortic segment as a result of CPR in up to 1% of resuscitated instances, with a higher incidence of this injury observed with the utilization of cardiopumps [17]. Previous research has identified serious intra-thoracic injuries in 42% of individuals following unsuccessful CPR. These injuries could contribute to the death or may be potentially lethal, given that the return of spontaneous circulation had been achieved. The combination of an aortic tear with concomitant rupture of solid abdominal organs (liver and spleen) is rare and represents exceptionally severe blunt force trauma delivered during resuscitation efforts. In addition, injuries to the abdominal organs, particularly the spleen and liver, are also encountered during indirect cardiac massage. The left lobe of the liver is the most commonly injured organ, while the right lobe, spleen, and stomach are less commonly injured. In his study, Krischer observed traumatic changes in the liver in the form of ruptures or subcapsular hematomas in 2.1% of cases, whereas he observed gastric injuries in only 1% of cases. The fact that the deceased had consumed alcohol prior to collapse is noteworthy. Alcohol consumption, particularly when combined with pre-existing, severe, undiagnosed cardiovascular disease like coronary artery hypoplasia and atherosclerosis, is a known risk factor for triggering acute cardiac arrhythmias and sudden cardiac death. This factor supports the determination of a natural underlying cause of collapse (acute coronary failure). Furthermore, the initial CPR was performed by her intoxicated boyfriend (blood alcohol level of 2.54 g/kg), which likely contributed to the "vehement" nature of the initial resuscitation and the severity of the subsequent CPR-associated injuries [1-4,16,18-20]. The issue of CPR-associated injuries (prevalence, risk factors, types of injuries, and mechanisms of their occurrence) is a significant area from a forensic point of view. Especially, CPR-associated injury classified as life-threatening or lethal presents a considerable problem. From a forensic perspective, CPR-associated injuries - particularly those classified as life-threatening or lethal - constitute a crucial area of investigation. The forensic pathologist must address the following two primary considerations during autopsy: (i) the necessity of distinguishing injuries caused by CPR from those resulting from blunt force trauma and (ii) the determination of whether CPR-associated injuries contributed to or caused the death of the resuscitated person. To address the second point, it is critical to establish whether the CPR-associated injuries occurred before or after the individual's death. Suppose the autopsy clearly establishes another unambiguous cause of death separate from the potentially lethal CPR-associated injuries. In that case, the conclusion is straightforward, i.e., the CPR injuries did not impact the person’s health or contribute to their death. In the present case, the young woman had undergone very vigorous CPR following cardiac arrest. Autopsy revealed a complex of fatal injuries, including aortic rupture, pulmonary contusion, hemothorax, liver rupture, splenic rupture, and hemoperitoneum. From a forensic standpoint, these represented high-intensity blunt force injuries, which, given the post-mortem examination and scene circumstances, were attributed to vigorous CPR. The key investigative question was whether CPR resulted in the return of spontaneous circulation (ROSC) and whether the severe traumatic injuries subsequently caused the young woman's death. Although there was no other unambiguous traumatic cause of death, the autopsy revealed natural diseases (superficial atherosclerosis and hypoplasia of the right and left circumflex coronary arteries) leading to the development of acute coronary failure.

Of note, the determination of a natural (non-traumatic) cause of death was primarily based on the confirmed lack of signs of vital response to the severe resuscitation injuries. Specifically, only 100 mL of blood was found in the pleural cavities (despite the aortic rupture), and only 400 mL of blood was found in the abdominal cavity (despite the liver and splenic ruptures). This low volume of hemorrhage, combined with the definitive histopathological evidence demonstrating the absence of vital reaction at the margins of the injuries, leads us to conclude that the vigorous CPR did not achieve sustained ROSC. Thus, the fatal CPR-related injuries are highly suggestive of having developed post-mortem. We concluded that the vigorous CPR did not achieve ROSC, and thus, the fatal CPR-related injuries most likely developed after the young woman’s death.

## Conclusions

The forensic pathologist must definitively determine whether death resulted from natural causes or from CPR-related injuries. In this specific case, the cause of death was ascertained to be natural, based on the circumstances of sudden collapse, the presence of coronary artery hypoplasia and atherosclerosis, and, critically, compelling histological evidence demonstrating the absence of signs of a vital reaction to the CPR-associated injuries. It was concluded that the fatal CPR-associated injuries are highly suggestive of having developed post-mortem. This case emphasizes the necessity of detailed histopathological assessment to differentiate potentially lethal post-mortem CPR trauma from trauma sustained while circulation was viable, providing a critical tool for forensic practitioners.
